# Leptin and fractalkine: novel subcutaneous cytokines in burn injury

**DOI:** 10.1242/dmm.042713

**Published:** 2020-04-29

**Authors:** Dominic Friston, Sini Junttila, Julia Borges Paes Lemes, Helen Laycock, Jose Vicente Torres-Perez, Elizabeth Want, Attila Gyenesei, Istvan Nagy

**Affiliations:** 1 Nociception Group, Section of Anaesthetics, Pain Medicine and Intensive Care, Department of Surgery and Cancer, Imperial College London, Chelsea and Westminster Hospital, 369 Fulham Road, London SW10 9NH, UK; 2 Bioinformatics and Scientific Computing, Vienna Biocenter Core Facilities, Dr. Bohr-Gasse 3, 1030 Vienna, Austria; 3 Department of Structural and Functional Biology, Institute of Biology, State University of Campinas, Campinas, Carl Von Linnaeus, Sao Paulo, 13083-864, Brazil; 4 Department of Metabolism, Digestion and Reproduction, Imperial College London, Exhibition Road, London SW7 2AZ, UK; 5 Department of Physiology, University of Debrecen, Debrecen, Nagyerdei krt 98, H-4012, Hungary

**Keywords:** Skin, Leptin, Fractalkine, Cytokines, Burn, Microdialysis

## Abstract

Burn injury is a pathology underpinned by progressive and aberrant inflammation. It is a major clinical challenge to survival and quality of life. Although the complex local and disseminating pathological processes of a burn injury ultimately stem from local tissue damage, to date relatively few studies have attempted to characterise the local inflammatory mediator profile. Here, cytokine content and associated transcriptional changes were measured in rat skin for three hours immediately following induction of a scald-type (60°C, 2 min) burn injury model. Leptin (*P*=0.0002) and fractalkine (*P*=0.0478) concentrations were significantly elevated post-burn above pre-burn and control site values, coinciding with the development of burn site oedema and differential expression of leptin mRNA (*P*=0.0004). Further, gene sequencing enrichment analysis indicated cytokine-cytokine receptor interaction (*P*=1.45×10^−6^). Subsequent behavioural studies demonstrated that, following subcutaneous injection into the dorsum of the paw, both leptin and fractalkine induced mechanical allodynia, heat hyperalgesia and the recruitment of macrophages. This is the first report of leptin elevation specifically at the burn site, and the first report of fractalkine elevation in any tissue post-burn which, together with the functional findings, calls for exploration of the influence of these cytokines on pain, inflammation and burn wound progression. In addition, targeting these signalling molecules represents a therapeutic potential as early formative mediators of these pathological processes.

## Abstract (precis)

**Summary:** Burn injury is associated with increased levels of leptin and fractalkine in the skin. Signalling by these cytokines leads to macrophage accumulation and hypersensitivity to heat and mechanical stimuli.

## INTRODUCTION

Burns, one of the most common causes of traumatic tissue injury, are characterised by tissue denaturation with subsequent development of a local inflammatory response that can persist for weeks (Laycock et al., 2013; Jeschke et al., 2007; Finnerty et al., 2006). As with other major traumas, the local inflammatory response can ‘spill’ beyond the burn site and may trigger the development of a systemic inflammatory state (manifesting as sepsis or systemic inflammatory response syndrome; Schulte et al., 2013; Constantian, 1978; Lenz et al., 2007) and potentially multiple organ dysfunction syndrome (Beal and Cerra, 1994; Lenz et al., 2007). Systemic inflammatory states predict mortality following burn injury (Goodarzi et al., 2014; Williams et al., 2009) and remain high priority targets for clinical intervention.

The local inflammatory response at the burn injury site relies on the production and release of a range of inflammatory mediators which are pivotal for the progression of the wound and drive the development of systemic effects (Shupp et al., 2010). Cytokines are particularly important in the pathogenesis of inflammatory states (Bone, 1996; Billingham, 1987) and several, including tumour necrosis factor alpha (TNF-α) and interleukin-1β and -6 (IL-1β and IL-6; Drost et al., 1993, de Bandt et al., 1994), are known to be elevated in burn tissue. However, as-of-yet unidentified cytokines may significantly contribute to the development of both the local and systemic inflammatory response in burn injury.

Although burn injury is a temporally dynamic pathology with distinct local and systemic manifestations, few studies have simultaneously sampled both locally and continuously from the burn site. Typically, investigations specifically targeting the experimental site have employed destructive methods limited to measuring single points in time or, alternatively, continuous data collection has been prioritised at the expense of specificity to the burn site via collection of systemic samples such as serum. To expand understanding of the cytokine profile of the burn injury extracellular milieu, we combined an established deep partial-thickness rodent model of scald-type burn injury with the novel application of subcutaneous microdialysis, a sampling method which is both continuous and specific to the microdialysis site (White et al., 2011; Torres-Pérez et al., 2017; Friston et al., 2019). This enabled the time series analysis of the availability of 27 cytokines via a multiplex rat cytokine/chemokine assay. The findings were supported by RNA next-generation sequencing of skin samples excised from the microdialysis sites, performed to screen for altered expression profiles of genes related to cytokine synthesis and signalling and functional assessments.

## RESULTS

The burn model induced erythema (not shown) and the development of oedema (Fig. S1A-D). Histological examination revealed the disruption of the structure of the epidermis and dermis by burn injury, the latter becoming gradually swollen and filling with eosinophilic material over time (Fig. S1A-C). Nuclei became pycnotic and, at 3 h post-burn, signs of karyorrhexis were evident (Fig. S1C). Blood vessels were dilated or destroyed (Fig. S1A-C).

**Fig. 1.**
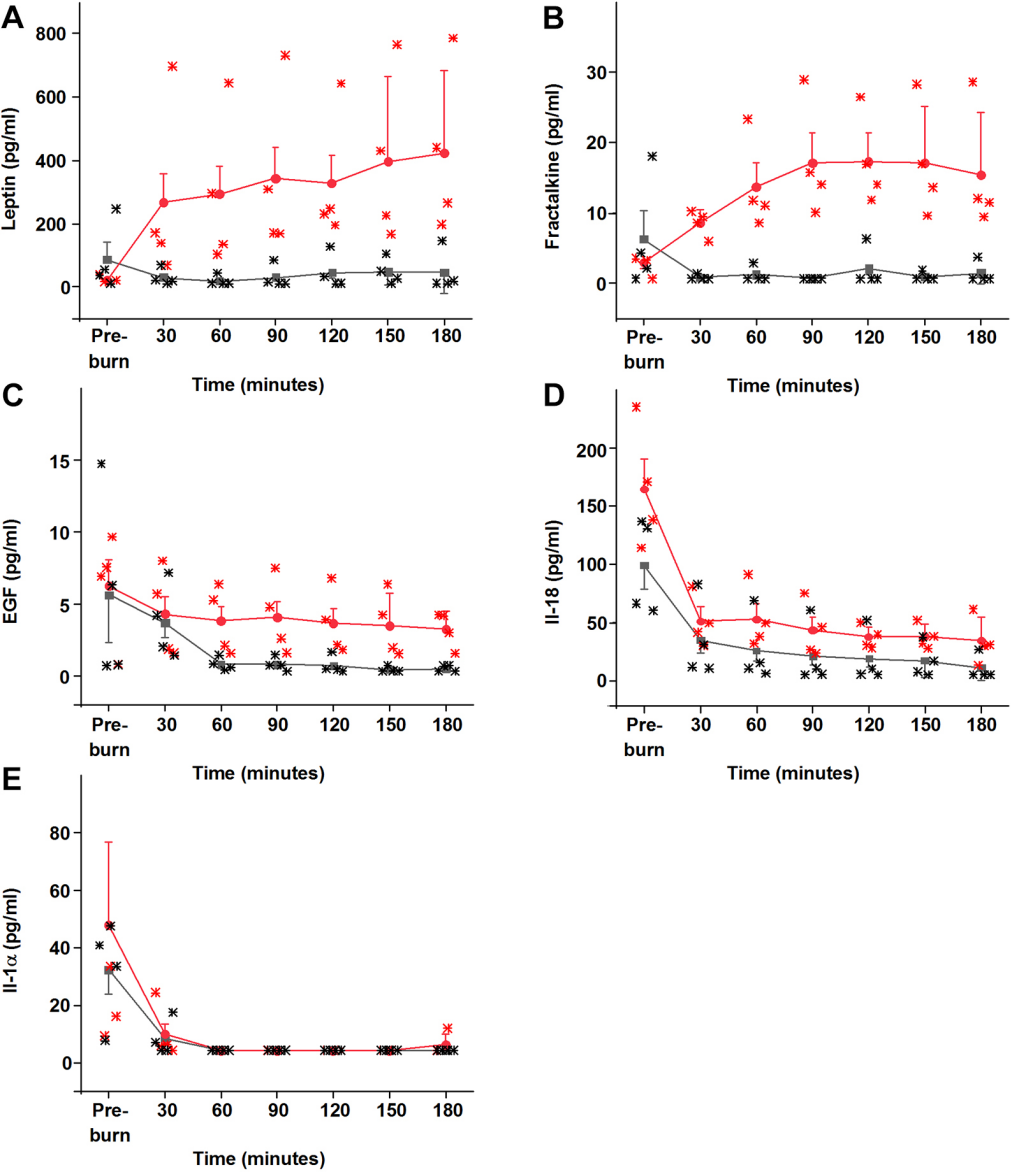
**Burn injury induces the subcutaneous accumulation of various cytokines/chemokines.** (A-E) Time course of changes in five cytokines in the ipsilateral (red) and contralateral (black) sides. Leptin (A) and fractalkine (B) were both significantly elevated post-burn, whereas the increased concentrations of GM-CSF and eotaxin were not statistically significant (not shown). The analysis indicated that the higher mean post-burn EGF concentrations in the burn site were consistent with pre-burn measurements and were not an effect of the burn (C). IL-18 (D) and IL-1α (E) were significantly elevated pre-burn at both burn and control microdialysis sites, presumably as a result of probe insertion, and recovered without being affected by the burn itself. Data are mean±s.e.m. Significance was set at *P*<0.05 following FDR-correction. Two-way ANOVAs and Grubb's tests were performed with Origin 9.1 and the Benjamini–Hochberg procedure was performed in Matlab R2014a. *n*=4.

Among the 27 cytokines included in the panel, 11 had a sufficient number of measurements at detectible concentrations for meaningful comparison and were brought forward for analysis (Table 1). The interstitial concentrations of two cytokines, leptin [*F*(1,52)=24.20935, false discovery rate (FDR)-corrected *P*=0.0002] and fractalkine [*F*(1,52)=7.04694, FDR-corrected *P*=0.0478; Fig. 1A,B], were significantly elevated post-burn (as indicated by significant interaction effects between microdialysis site and time; Table 1). Both leptin and fractalkine exhibited similar time courses, consisting of rapid and sustained increases in concentration specific to the burn site and exceeding levels recorded pre-burn (Fig. 1A,B). It was determined that the higher burn site recordings of epidermal growth factor (pro-epidermal growth factor; EGF) (Fig. 1C) resulted from differences in the tissue before induction of the model rather than the burn, despite the similarity in pre-burn mean concentrations, as indicated by the significant site effect [*F*(1,52)=9.29601, FDR-corrected *P*=0.0238; Table 1]. IL-18 and IL-1α concentrations were elevated (Fig. 1D,E) by probe insertion at both the burn and control microdialysis sites [as indicated by significant effects of time: IL-18, *F*(1,52)=22.81937, FDR-corrected *P*=0.0002; IL-1α, *F*(1,52)=59.02708, FDR-corrected *P*<0.0001]; these transient increases in concentrations appeared to resolve quickly and were not affected by the burn.

**Table 1.**
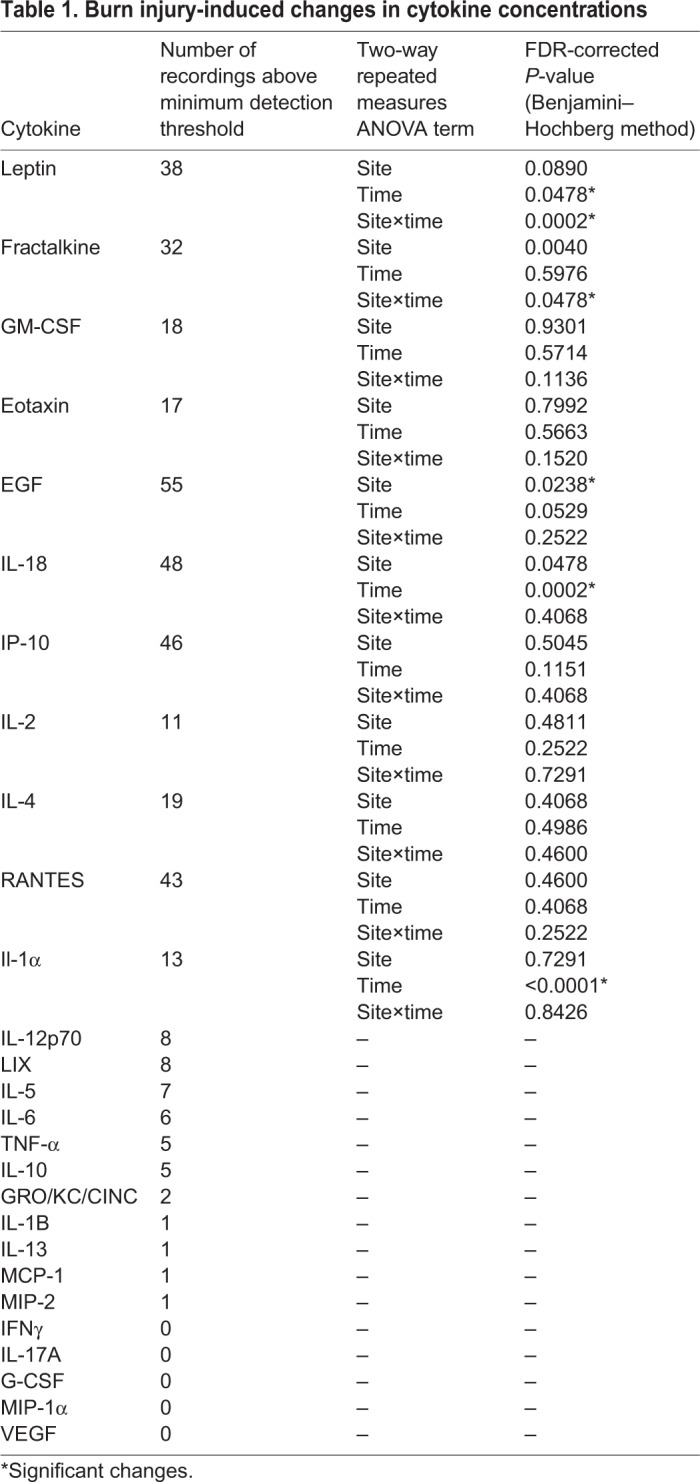
**Burn injury-induced changes in cytokine concentrations**

**Fig. 2.**
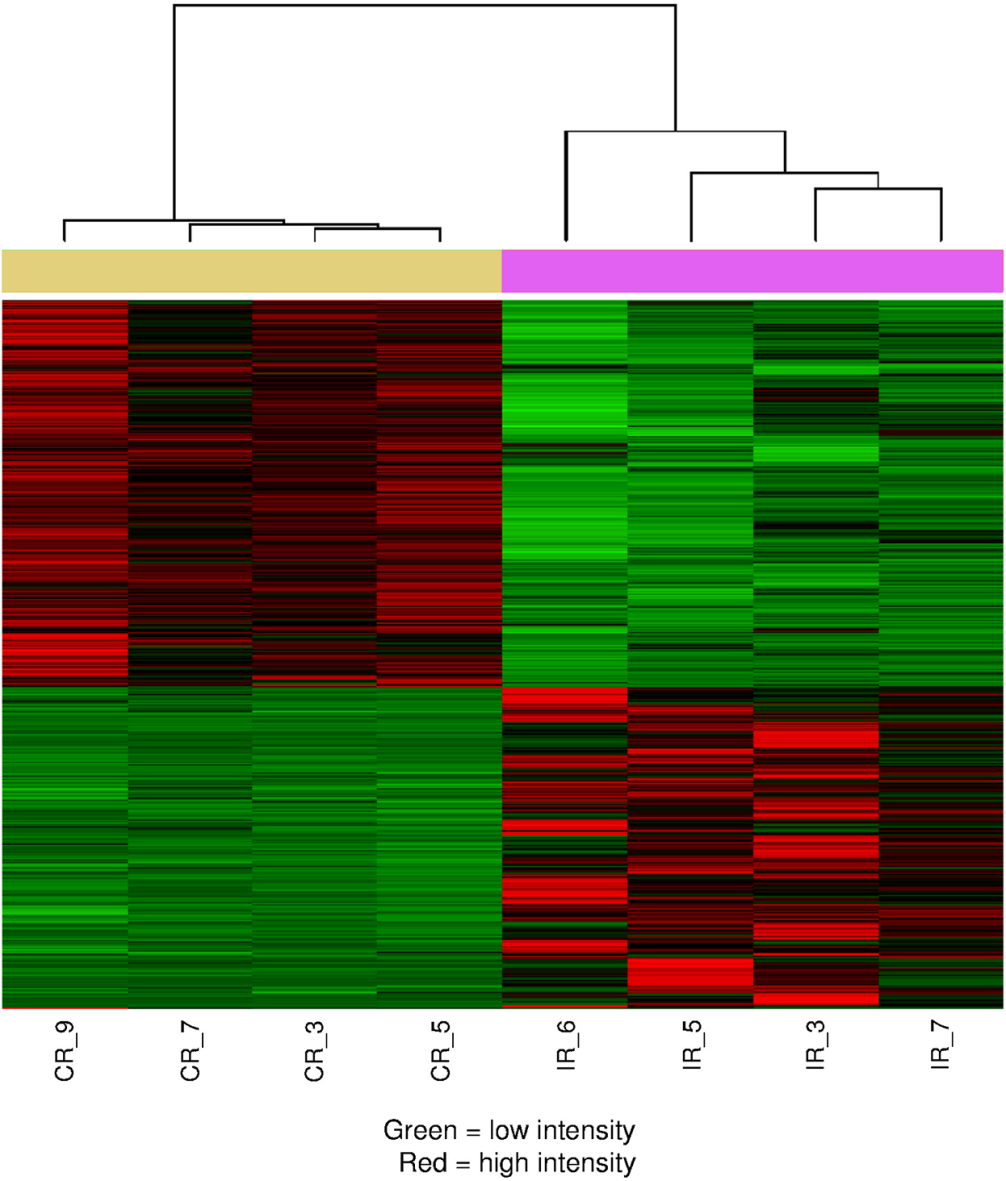
**Burn injury induces significant changes in gene expression in the skin.** mRNA isolated from four ipsilateral (IR_6, IR_5, IR_3 and IR_7) and four contralateral (CR_9, CR_7, CR_3 and CR_5) skin samples was sequenced. mRNA samples are arranged in columns. Genes that are significantly differentially expressed between burn and control samples are arranged, based on the expression level, in rows of the heat map; green indicates low expression level, whereas red indicates high expression level. The contralateral (control) samples are indicated by the yellow bar above the heat map, and the ipsilateral (burn) samples by the purple bar. The heat map shows that the expression of the genes identified as differentially expressed is high in the burn samples and low in the control samples or vice versa and that the four replicate samples in both the control group and the burn group have similar expression patters across the differentially expressed genes. The expression level of individual genes in each skin sample can be found in GEO dataset GSE102811.

Analysis of the RNA sequencing (RNA-seq) data detected the expression levels of 26,404 genes, with 2610 genes identified as differentially expressed between skin of the burn and control microdialysis sites. Analysis showed that 1186 genes were upregulated at the burn site, whereas 1424 genes were downregulated (Fig. 2; Table S1). Genes for the 11 cytokines undergoing statistical comparison were also identified in the RNA-seq data, but only two of them, leptin (*P*=0.0004) and RANTES (Ccl5; *P*=0.0015), were significantly different between burn and control samples (Table S1). Leptin expression was significantly lower at the burn site than the control, whereas RANTES was significantly upregulated (Table S1).

**Fig. 3.**
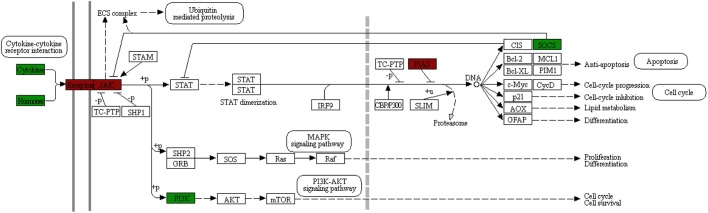
**KEGG map of the Jak-STAT signalling** **pathway.** Of the 110 genes annotated to this KEGG pathway, 27 were differentially expressed between burn and control microdialysis site RNA. Differentially expressed genes are labelled green for downregulation and red for upregulation.

**Fig. 4.**
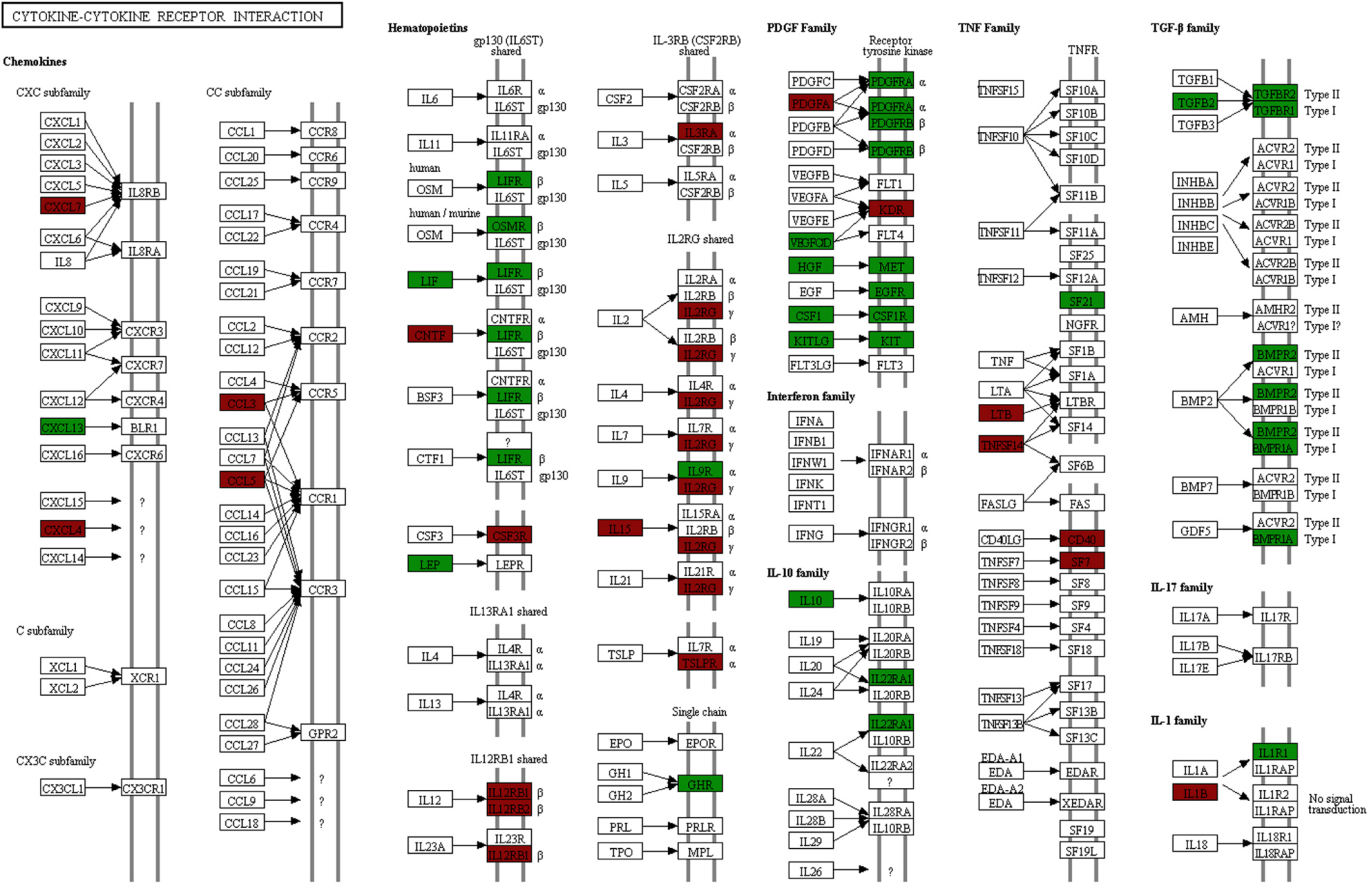
**KEGG map of the cytokine-cytokine interaction pathway.** Of the 156 genes annotated to this KEGG pathway, 48 were differentially expressed between burn and control microdialysis site RNA. Differentially expressed genes are labelled green for downregulation and red for upregulation.

Enrichment analysis of Kyoto Encyclopedia of Genes and Genomes (KEGG) pathways and Gene Ontology (GO) terms among the differentially expressed genes was also performed. The Jak-STAT signalling pathway and cytokine-cytokine receptor interactions were identified as significantly enriched KEGG pathways (Figs 3 and 4, respectively) and several cytokine-related GO terms, such as cytokine activity, cytokine binding and response to cytokine, were significantly enriched in the biological process and molecular function categories (Table S2).

**Fig. 5.**
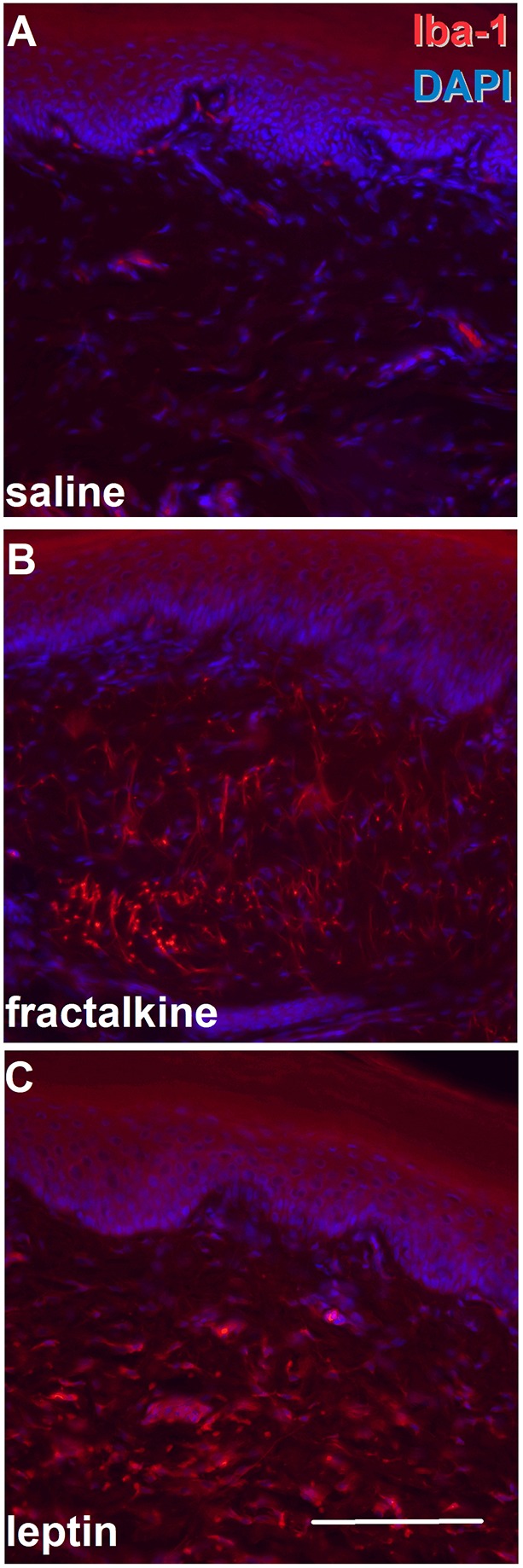
**Both leptin and fractalkine induce macrophage accumulation in the skin.** (A-C) Saline (A), fractalkine (B; 100 ng/50 µl) or leptin (C; 500 ng/50 µl) were injected into the hind paw and paw skin dissected 2 h, 2.5 h and 2 h after injection, respectively. Sections were then reacted with an anti-Iba1 antibody. Although in the saline-injected skin very few Iba1-immunopositive structures (red) are seen, both fractalkine and leptin injection resulted in a significant increase in the Iba1-immunopositive profiles. DAPI (blue) indicates nuclei. Scale bar: 100 µm.

In functional studies, first we assessed the effect of leptin and fractalkine on macrophage accumulation. In control, saline-injected skin sections exhibited very few structures immunopositive for the ionised calcium binding adaptor molecule 1 (Iba1; Aif1) that identifies various immune cells including macrophages, monocytes and neutrophils (Fig. 5). Although double staining for identifying the immunopositive structures was not performed, their morphology and proximity to capillaries indicated that they were monocytes or neutrophils. In contrast, a significant number of immunopositive structures were observed in skin sections from both fractalkine- or leptin-injected rats, with fractalkine eliciting a particularly strong immunostaining (Fig. 5). Based on their morphological appearance (e.g. having processes and forming a ‘network’), these stained cells were identified as macrophages (Fig. 5).

**Fig. 6.**
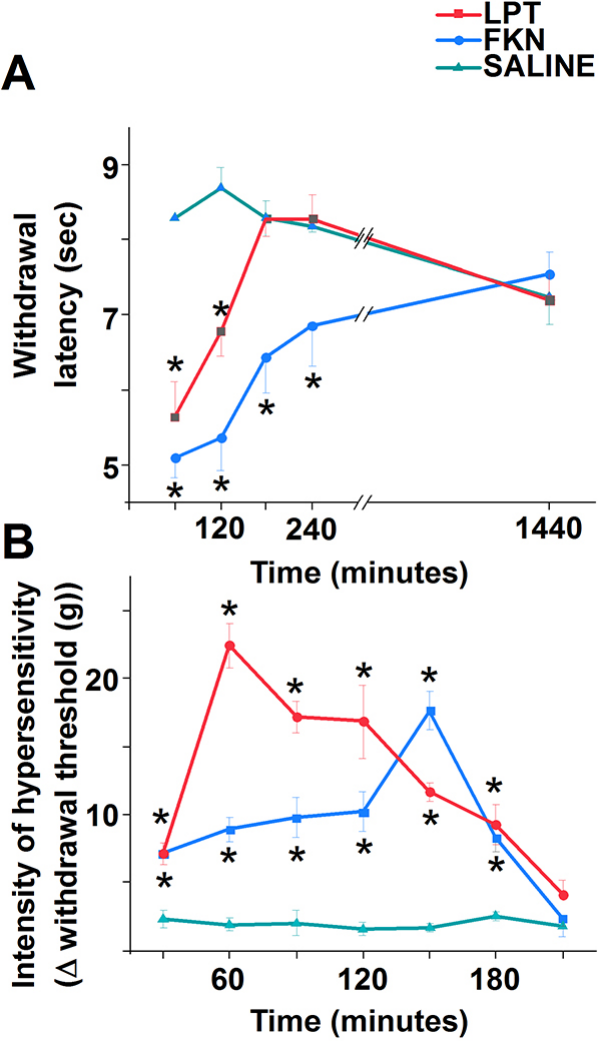
**Both leptin and fractalkine induce hypersensitivity to both heat and mechanical stimuli.** (A,B) Leptin (red; 500 ng/50 µl), fractalkine (blue; 100 ng/50 µl) or saline (green) was injected into the hind paw, and responses to heat (A), using a hot plate, and mechanical stimuli (B), using electronic von Frey apparatus, were assessed. Both leptin and fractalkine induced hypersensitivity to both kinds of stimuli, though with differing time courses. Data are mean±s.e.m. Asterisks indicate significant differences from control at *P*<0.05; two-way ANOVA with Bonferroni post-hoc test. *n*=5 in each experiment.

Next, we assessed how subcutaneous injection of leptin and fractalkine affects pain-related behaviour. In register with the observed macrophage recruitment, both leptin and fractalkine induced the development of mechanical allodynia and heat hyperalgesia (Fig. 6), although the temporal profiles of these responses varied. Firstly, the development of mechanical allodynia induced by leptin progressed faster than that of fractalkine (Fig. 6A), though mechanical stimulation-evoked pain-related behaviour returned to baseline levels by 3.5 h post-injection in either case (Fig. 6A). Secondly, the development of heat hyperalgesia progressed equally rapidly for both leptin and fractalkine, though the fractalkine-induced reduction in paw withdrawal latency persisted for a longer duration (Fig. 6B). These differences imply the recruitment of distinct pronociceptive mechanisms by either cytokines in each pain modality.

## DISCUSSION

### Microdialysis aids the elucidation of the dynamics of local pathophysiology in burn injury

Burn injury sampling studies typically compromise either data specificity to the burn site or the temporal resolution; most sampling methods such as skin biopsies are destructive or invasive enough to preclude continuous sampling. Conversely, systemic levels of analytes measured in blood samples are not representative of the local pathology. The collection of local and continuous longitudinal data is important for elucidating the dynamics of the pathophysiology of burn injury. This study demonstrates the viability of microdialysis, which allows continuous sampling specifically from the burn site. Further, samples are suitable for multiplex cytokine profiling.

Using this method we found two cytokines, leptin and fractalkine, significantly elevated in the subcutaneous tissue of the rat paw following a deep partial-thickness burn injury. Although leptin levels have been shown to be elevated in the serum of burn-injured patients (Abdel-Hafez et al., 2007), the present study is the first to report increased local interstitial leptin levels in burn injury. To the best of our knowledge, this study is also the first to report increased expression of fractalkine in any tissue following burn injury.

### Leptin significantly contributes to the local pathophysiology of the burn injury via multiple mechanisms

Although leptin concentration is significantly increased in the interstitium, leptin mRNA expression exhibits a significant decrease when the entire injured skin is assessed. This discrepancy suggests that dying cells, particularly adipocytes, which are the primary site of leptin synthesis (Cammisotto et al., 2006), may be the source of leptin in the interstitium for at least the first 3 h of burn injury. However, owing to variable regulatory mechanisms, translational efficiencies and mRNA and protein half-lives, mRNA and protein level correlation is notoriously poor (Maier et al., 2009). Further, although we assessed the interstitial protein abundance continuously, the mRNA expression of leptin represents a ‘snapshot’ at the 3 h post-injury time-point. Hence, a transient increase in leptin mRNA level may have gone undetected. Nonetheless, the differential mRNA expression detected for leptin was among the most significant in the gene sequencing analysis, whereas leptin was the most significantly altered cytokine at protein level. These findings together indicate that leptin is not just an ‘accidental passive bystander’ in the local pathophysiological processes in burn tissue. This view is supported by a series of changes in the RNA expression of leptin downstream effectors in the burn-injured skin. Firstly, the expression of mRNA for leptin signalling molecules janus kinase 2 (JAK2) and signal transducer activator of transcription 3 (STAT3; Vaisse et al., 1996; Frank et al., 2000; Sano et al., 1999) is significantly reduced, whereas that of JAK3 is significantly increased following burn injury. Secondly, the expression of genes encoding NF-κB, Bcl-x_L_ (Bcl2l1) and T-bet (Tbx21), proteins involved in leptin-induced T cell polarisation toward the Th1 phenotype (Mattioli et al., 2005; Batra et al., 2010; Fujita et al., 2002), are upregulated by our burn model. Thirdly, mRNA expression for several leptin-regulated cytokines, including IL-1β and interferon gamma, exhibit significant/near significant increases following burn injury.

Previous data also support the significant contribution of leptin, which is principally known for its roles in regulating appetite and energy expenditure (Ahima et al., 1996), to both the local and systemic pathophysiology of burn injury. Leptin has been shown to play a significant role in inflammatory responses after burn injury (Matarese et al., 2005; Sarraf et al., 1997; Faggioni et al., 2001). Further, leptin indicates and regulates several pathological processes that are characteristic features in burn injury, including metabolic demand (Wade et al., 2013), hypovolaemia (Mark et al., 1999) and surgical stress (Ahima et al., 1996). These effects may be exerted by regulating the chemotaxis (Faggioni et al., 2001), cytokine release (Martín-Romero et al., 2000; Mattioli et al., 2005; Wong et al., 2007) or other mechanisms (Najib and Sánchez-Margalet, 2002; Sanchez-Pozo et al., 2003; Maingrette and Renier, 2003; Zarkesh-Esfahani et al., 2004; Matarese et al., 2005; Lord et al., 1998) of various immune cells. In addition to pro-inflammatory functions, leptin may also have beneficial effects in burn injury as it has been suggested to promote survival in acute sepsis (Correia et al., 2001), attenuate the inflammatory response and multiple organ failure (Çakir et al., 2005) and promote wound healing (Sano et al., 1999). Leptin has been implicated in post-burn angiogenesis (Sierra-Honigmann et al., 1998; Bouloumié et al., 1998), keratinocyte proliferation (Frank et al., 2000) and mitogenic action on a variety of cells required for tissue repair (Bouloumié et al., 1998; Gainsford et al., 1996).

Beyond these beneficial effects, leptin has also been implicated in the development and maintenance of burn-induced tactile allodynia (Maeda et al., 2009). This is consistent with our present finding of a rapid onset of mechanical allodynia following subcutaneous leptin injection. Interestingly, macrophage stimulation and/or accumulation, reported in the present study, has been shown to underlie this pro-algogenic effect (Maeda et al., 2009). We also report the development of heat hyperalgesia after subcutaneous leptin injection. The fast onset of leptin-evoked pain-related behaviour suggests that the effect could be at least partially mediated by leptin receptors expressed on primary sensory neurons (Alvarez et al., 2014; Chen et al., 2006). However, in addition to these local effects, leptin can be transported to the central nervous system (Banks et al., 1996) where it upregulates spinal expression of the N-methyl-D-aspartate receptor, playing a crucial role in the development of central sensitisation underlying the development of persistent pain (Lim et al., 2009).

### Fractalkine contributes to the local pathophysiology of burn injury

Fractalkine is produced by monocytes, macrophages, fibroblasts, endothelial and dendritic cells in inflammatory conditions (Ruth et al., 2001), and is expressed locally by various cells including keratinocytes, Langerhans cells, fibroblasts and endothelial cells. However, the anti-fractalkine antibody identifies only the soluble form of fractalkine that is formed when the membrane-bound fractalkine is cleaved from its transmembrane domain (Umehara et al., 2004; Garton et al., 2001). Judging by the histological appearance of the injured skin, the majority of the cells, which potentially could be the source of fractalkine, may not survive the burn. Yet, although the concentration of fractalkine is significantly increased in the interstitium, the fractalkine mRNA is not significantly changed in the injured skin. Hence, a proportion of fractalkine found in the interstitium could be cleaved from fractalkine-synthesising cells invading the tissue.

Here, we demonstrate that subcutaneous injection of fractalkine induces recruitment of macrophages. Further, we report that fractalkine injection into the paw results in the development of both mechanical allodynia and thermal hyperalgesia. The fractalkine receptor has been found on a subpopulation of primary sensory neurons in which it induces excitatory effects, though the exact subpopulation of the neurons has not been identified (Oh et al., 2001). In combination, these data suggest that at least a proportion of the fractalkine-induced pain-related behaviour is mediated by direct effect of this cytokine on primary sensory neurons.

Soluble fractalkine functions as a chemoattractant for monocytes, natural killer (NK) cells and T cells (Bazan et al., 1997). The chemotactic function of fractalkine appears to regulate a myriad of physiological and pathological processes such as adaptive immunity, cardiac failure, angiogenesis and the development of pain (Ruth et al., 2001; Yoneda et al., 2000; Umehara et al., 2004; Jones et al., 2010; Ikejima et al., 2010; Kimouli et al., 2009). Hence, the local increase in fractalkine may have both beneficial and detrimental effects on burn tissue. However, the apparent functional versatility of those effects are not clear, and this calls for further study of the role and potential therapeutic manipulation of this chemokine in burn injury.

### The expression of other cytokines identified by the panel is not affected by burn injury

Two cytokines of the IL-1 family, IL-18 and IL-1α, exhibited statistically significant increases in their concentrations in the pre-injury samples compared to their respective concentrations found in post-burn samples. It appears that tissue injury resulting from the insertion of the probe, but not burn injury, induced the release of these cytokines. Although this elevation of IL-1α is consistent with its abundance in the epidermis and release upon damage (Wood et al., 1996; Cohen et al., 2010), the lack of its increase after the burn is perplexing, as IL-1α has been considered a damage-associated molecular pattern molecule (Jensen, 2010). A possible explanation for this discrepancy could be that, although IL-1α is released in necrotic conditions, during apoptosis it concentrates in dense nuclear foci, is rendered less mobile and is not released with the other cytoplasmic contents (Cohen et al., 2010). Although the relative contribution of apoptosis and necrosis in the early stages of burn injury is poorly understood, the IL-1α concentration profile found in the present study suggests that cell death remains apoptotic for at least 3 h following the insult.

Although the panel we used contained antibodies against cytokines previously reported to be elevated in burn injury, including some by the action of leptin (Wong et al., 2007; Martín-Romero et al., 2000), none of these exhibited an increase in the present study. Many were not detected in the samples despite the sensitivity of the assay. While it is possible that a burn effect was present but undetectable, the lack of detection would not necessarily be inconsistent with previous burn injury studies. The overwhelming majority of previous reports show the concentrations of these analytes in systemic circulation (Drost et al., 1993; Liu et al., 1994; Biffl et al., 1996; Kowal-Vern et al., 1994; Rodriguez et al., 1993; Struzyna et al., 1995; Yeh et al., 1999) rather than locally at the burn site. Local sampling methods implicating these cytokines, such as the use of homogenised skin (Ipaktchi et al., 2006), adipose tissue lysate (Saraf et al., 2016) and blister fluid (Rhodes et al., 1999; Ono et al., 1995), may not represent the subcutaneous interstitium as accurately as microdialysis. Further, the analyte elevations reported typically occur over several days rather than in the hours immediately following the initial trauma, which were the focus of this study; this initial period following injury remains particularly poorly documented. Previous applications of microdialysis to burn tissue sampling implicated IL-7 and IL-13 over a time-frame far exceeding the duration of the present report (Angst et al., 2008) and the acute phase response to which IL-1β, IL-6 and TNF-α contribute also far exceeds the 3 h modelled presently (Colley et al., 1983). The non-destructive nature of microdialysis and the short duration of the present experiment may therefore explain the lack of a burn effect among previously implicated analytes included in the panel, but may further highlight leptin and fractalkine among the early formative mediators of the inflammatory response in burn injury.

In conclusion, we have identified leptin and fractalkine as two novel local inflammatory mediators in burn injury. These cytokines have various potential roles in burn pathology, principally relating to the immune system and inflammation. In addition, we have demonstrated that both leptin and fractalkine are likely to contribute to the development of burn injury-associated pain, though at present it is not clear whether or not they act through the principal noxious stimuli-sensing ion channels such as transient receptor potential ion channel, vanilloid subfamily member 1 (Caterina et al., 1997), 2 (Ahluwalia et al., 2002) and 4 (White et al., 2016), or ankyrin subfamily member 1 (Sousa-Valente et al., 2014), which are likely to be involved in the very early components of pain. Nevertheless, given the destructive, inflammatory nature of burn injury, their potential as targets for therapeutic intervention should be investigated. In addition, we encourage further application of techniques enabling evaluation of the temporally dynamic progression of the burn injury, with spatial specificity, to elucidate the pathology of this complex trauma.

## MATERIALS AND METHODS

### Animals

All procedures were performed in accordance with the UK Animals (Scientific Procedures) Act 1986, the revised National Institutes of Health Guide for the Care and Use of Laboratory Animals, the Directive 2010/63/EU of the European Parliament and of the Council on the Protection of Animals Used for Scientific Purposes and the guidelines of the Committee for Research and Ethical Issues of the International Association for the Study of Pain. Good Laboratory Practice and ARRIVE guidelines were observed and all animal procedures were approved by veterinary services (Central Biological Services) at Imperial College London, UK. Every effort was taken to minimise the number of animals used.

Male Sprague-Dawley rats (125-200 g) were housed in climate-controlled rooms on a 12 h light/dark cycle and with food and water *ad libitum*. They were anaesthetised with 1.5 g/kg urethane by intraperitoneal (IP) injection. Body temperature was monitored and maintained at 37°C with a heat blanket, rectal thermometer and homeothermic control unit (50-7061-F; Harvard Apparatus). Four rats were used for microdialysis and subsequent gene expression studies. Paw circumference measurements and skin sections for histological assessments were collected from five additional rats used in a separate study (Torres-Pérez et al., 2017). For behavioural studies 15 rats were used.

### Microdialysis

We performed microdialysis as described previously (Friston et al., 2019). Briefly, sterile linear microdialysis probes (400 μm 3 MDa cut-off microdialysis catheters; Dermal Dialysis) were inserted into subcutaneous tissue of the dorsal aspects of both hind limbs. The probes were placed just below the dermis. The active uptake length of the probes (i.e. the length of the probes through which molecules from the interstitium could enter the perfusate) was 10 mm. Probe insertion was performed 50 min before the burn injury to allow for 20 min of fluidics equilibration and a further 30 min ‘flush’ period (Fig. S2). The latter enabled comparison of pre-burn cytokine levels within each animal and further allowed stabilisation of any inflammatory response resulting from probe insertion. The probes were connected to a Model ‘22’ syringe pump (Harvard Apparatus) using plastic tubes and Ringer's solution (Baxter) was perfused through the tubes and connected probes at a rate of 2 μl/min. Microdialysate was collected for 0.5 h pre-burn and 3 h post-burn in half hour fractions. Collected microdialysates were stored at −80°C until analysis.

### Burn model

A deep partial-thickness burn model was induced as described previously (Torres-Pérez et al., 2017; White et al., 2011). In brief, one hind paw was submerged into 60°C saline to the ankle for 2 min. The contralateral paw was simultaneously submerged in room temperature saline as a control. The circumferences of both the burn and control hind paws were measured at 5, 30, 60, 120 and 180 min post-injury. Upon completion of the experiments, animals were terminally anaesthetised with sodium pentobarbital (40 mg IP) and microdialysis sites were excised both to verify probe placement in subcutaneous tissue (confirmed on histology – data not shown) and for RNA extraction.

### Subcutaneous injection

Animals were randomly allocated into three different groups: the leptin group received a single injection of leptin, 500 ng/50 µl (Peprotech); the fractalkine group received a single injection of fractalkine, 100 ng/50 µl (Peprotech); the control group received the vehicle (0.9% NaCl/50 µl). The choice of leptin and fractalkine dose was based on previous studies and adapted to our route of administration, the subcutaneous tissue (Souza et al., 2013; Tian et al., 2011; Duan et al., 2007). Leptin, fractalkine or saline were injected using an insulin syringe (BD Ultra-Fine^®^, 29G) subcutaneously (SC) between the five callosities of the paw, the same region to which experimental mechanical and thermal stimuli were applied.

### Histology and immunostaining

Paw circumference measurements and tissues for histological verification of burn depth in the model were collected from five additional animals. These were transcardially perfused with physiological saline followed by 4% paraformaldehyde. Similarly, 2 or 2.5 h after leptin or fractalkine administration (time points correspond with the maximum algogenic effects) rats were also transcardially perfused with physiological saline followed by 4% paraformaldehyde. Skin was dissected, post-fixed in 4% paraformaldehyde for 4 h then transferred into 30% sucrose dissolved in 0.01 M phosphate buffer saline (PBS). For verification of burn depth, 14 μm sections were cut with a cryostat, stained with haematoxylin and eosin and examined with a light microscope (Leica).

Skin sections from saline-, fractalkine- or leptin-injected rats were processed for immunostaining. Briefly, sections were washed in PBS containing 0.3% Triton X-100 (PBS-T). Following incubation in 10% normal donkey serum (NDS) for 30 min, sections were incubated in an anti-Iba1 antibody (019-1974, batch 202212; FUJIFILM Wako Pure Chemical Corporation, raised in rabbit, 1:1000) diluted in PBS-T containing 1% NDS overnight at room temperature. These were visualised by incubation in donkey anti-rabbit IgG conjugated with Alexa Fluor 568 (1:1000, Invitrogen, A10042). Sections were washed in PBS-T containing 1% NDS three times for 10 min between incubations. Sections were covered using Vectashield (Vector Laboratories) and examined using a fluorescent microscope (Leica). Images taken by the Leica Application Suite software packages using a Retiga 2000R camera (QImaging) were not digitally modified beyond adjusting brightness and contrast or combining images captured with different excitation and emission filters.

### RNA extraction and next-generation sequencing

Following the conclusion of microdialysis, skin from both the burn and control sampling sites was excised and stored in RNAlater (Invitrogen). The tissue underwent rotor-stator homogenisation before RNA extraction conducted with RNeasy Fibrous Tissue Mini and Midi kits (Qiagen) according to the manufacturer's protocols.

Next-generation sequencing libraries were prepared from the isolated total RNA samples by poly-A enrichment (New England Biolabs) using the manufacturer's instructions. The samples were sequenced with HiSeq2500 (Illumina) on one lane using single-end sequencing with a 50 bp read length.

### Microdialysate analysis

The concentrations of 27 cytokines were measured in duplicate for each microdialysate fraction using the FlexMAP 3D (Luminex^®^) platform with a Milliplex Rat cytokine/chemokine panel (RECYMAG65K27PMX; Merck Millipore) according to the manufacturer's protocol. Sixteen cytokines, for which fewer than 20% of the duplicate recordings over all animals, experimental conditions and time points exceeded the assay's minimum detectible concentrations, were excluded from the analysis. For the remaining cytokines, undetectable concentration means were reassigned to the lower of either the lowest value recorded or the lower detection threshold reported by the plate reading xPONENT Software (Ver. 4.2 for FlexMAP 3D Technology). In order to avoid introducing artificially unequal variability in cytokine concentration across different time points (i.e. heteroscedasticity) during this process, variance within each condition was calculated and applied to any reassigned data around the required mean. A single outlier was detected in the eotaxin control pre-burn microdialysate by Grubb's test (maximum normed residual test) and was subsequently removed.

### RNA-seq data analysis

Inferred sequence of bases (reads) obtained from the next-generation sequencing run were mapped to *Rattus norvegicus* Rnor5.0 reference genome release with TopHat (version 2.0.9) (Kim et al., 2013). The read counts for each gene were detected using HTSeq (version 0.5.4p3) (Anders et al., 2015). The counts were normalised using the TMM normalisation from the edgeR package in R (McCarthy et al., 2012; Robinson et al., 2010). Enrichment analyses, identifying differentially represented networks of functionally associated genes, were performed using the topGO (http://www.bioconductor.org/packages/release/bioc/html/topGO.html) and gage packages in R. The RNA-seq data have been submitted to GEO with accession GSE102811 (Edgar et al., 2002; Barrett et al., 2013).

### Behavioural assessment

Behavioural assessment was conducted after SC leptin, fractalkine or vehicle injection. Mechanical nociceptive thresholds were measured by an automatic Electronic von Frey apparatus (Electronic Analgesimeter, Insight^®^) as described previously (Vivancos et al., 2004). The test consists of applying a von Frey hair tip to the central region of the paw with gradually increasing pressure controlled by a pressure transducer and computer. The stimulus ceases after the hind paw is withdrawn and the inducing force is recorded as the mechanical pain threshold. Rats were held in the experimental acrylic cages (12×20×17 cm) with wire grid floors for 30 min before testing to allow for acclimatisation. A mirror placed under the grid allowed a clear view of the rat hind paw. Animals were tested before SC injection and then subsequently every 30 min for a total of 210 min. Assessments at each time point included three measurements, which were then averaged. Results are expressed as the Δ mechanical threshold (g), calculated by subtracting the average of the last three measurements after the treatments from the average of the three measurements before treatments.

Heat hyperalgesia was assessed by measuring withdrawal time in response to a 45°C hot-plate (Insight^®^). Withdrawal latency was defined as the duration between the onset of the thermal stimulation and the animal responding either by jumping or licking the paw. Data are expressed as the averages of three measurements from each animal at 1, 2, 3, 4 and 24 h after SC injection of leptin, fractalkine or vehicle.

### Statistics

For statistical analysis of the microdiasylate readings, data were log transformed and burn and control cytokine concentrations were compared by two-way ANOVA [treatment (burn or control)×time (pre- or post-burn)]. The Benjamini–Hochberg procedure (Benjamini and Hochberg, 1995) was used for multiple testing correction and all *P*-values are listed with this correction applied. Significance was set at *P*<0.05 following false discovery rate (FDR)-correction. Two-way ANOVAs and Grubb's tests were performed with Origin 9.1 and the Benjamini–Hochberg procedure was performed in Matlab R2014a.

For statistical testing of gene expression changes, data were transformed using the voom method, which estimates the mean-variance relationship of the log-transformed read counts and generates a precision weight for each observation (Law et al., 2014). The differential expression between the burn and control samples was detected with the Limma package in R (Ritchie et al., 2015). Genes with fold change>2 and FDR<0.05 were identified as differentially expressed.

Results of behavioural experiments were analysed using two-way ANOVA followed by the Bonferroni post-hoc test. Differences were considered statistically significant at *P*<0.05. All data are mean±s.e.m.

## Supplementary Material

Supplementary information
