# Supplementary material for: Leptin and fractalkine: novel subcutaneous cytokines in burn injury
Source: Dis Model Mech. 2020 Apr 29;13(4):dmm042713. doi: 10.1242/dmm.042713 (PMC7197715; doi:10.1242/dmm.042713)
Supplement: Supplementary information [file dmm-13-042713-s1.pdf]

Supplementary Figure 1.

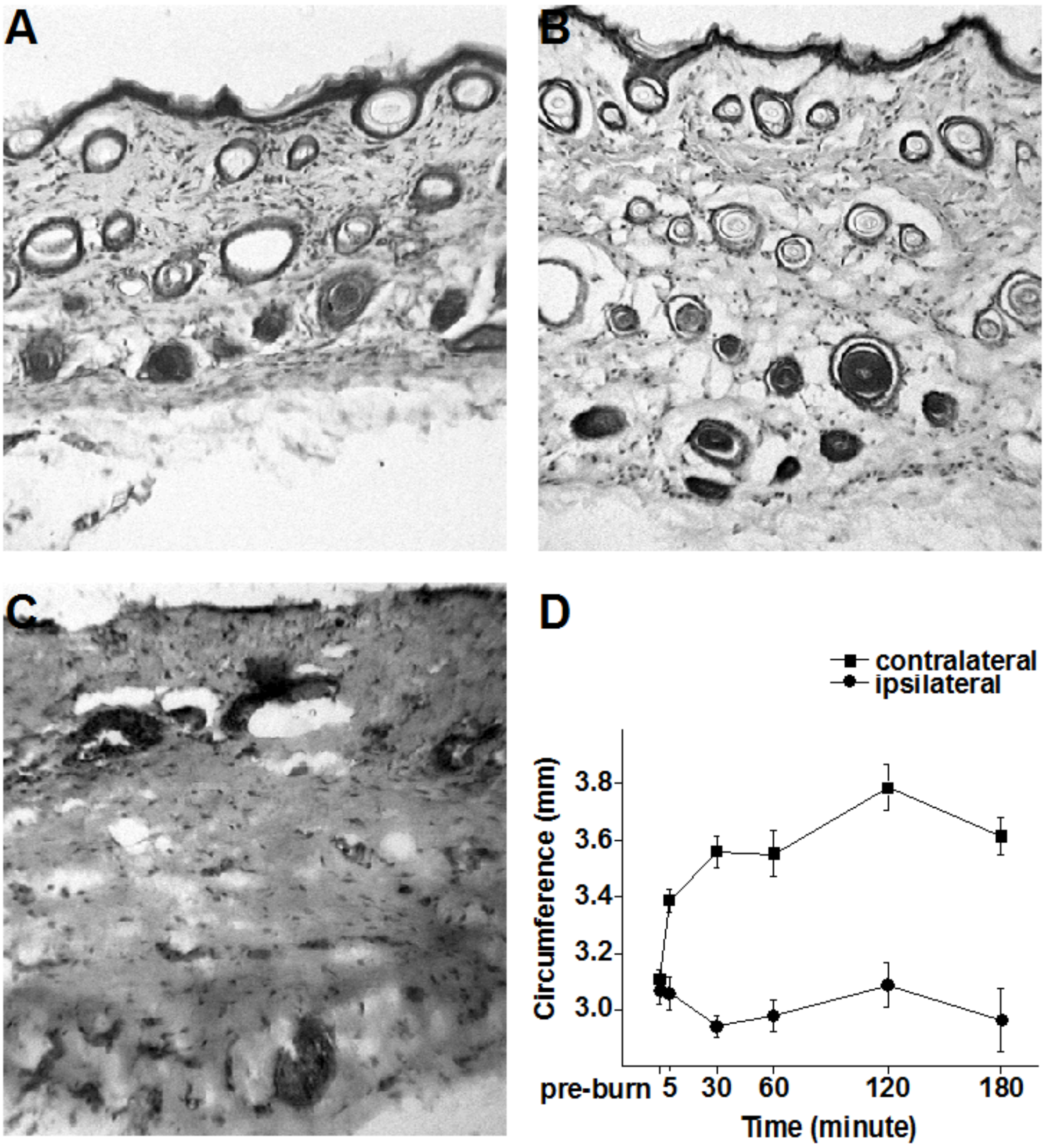

Figure S1. Our burn injury model is associated with the disruption of the skin structure and the development of oedema
(A) Microphotograph of a section cut from the dorsal aspect of a naive rat paw and stained with haemotaxilin-eosin. The image shows the normalstructure of the skin. (B) Microphotograph of a section cut from the dorsal aspect of a rat paw 5 minutes after the burn injury. The section was then stained with haemotaxilin-eosin. Note that the skin became oedematous and nuclei appear shrunken at this time point. However the structure (epidermis, dermis, hair follicules, etc) is still recognisable (C) Microphotograph of a section cut from the dorsal aspect of a rat paw three hours after burn injury. The section was stained with haemotaxilin-eosin. Note that the structure of the skin (both the epidermis and dermis) is destroyed; the basal layer of the epidermis is visible only in islands, blood vessels and hair follicles not recognisable, the entire tissue is filled with an eosinophyl material. Nuclei are picnotic and karriohexis is evident. (D) The line chart shows the development of oedema following the burn injury.

Supplementary Figure 2

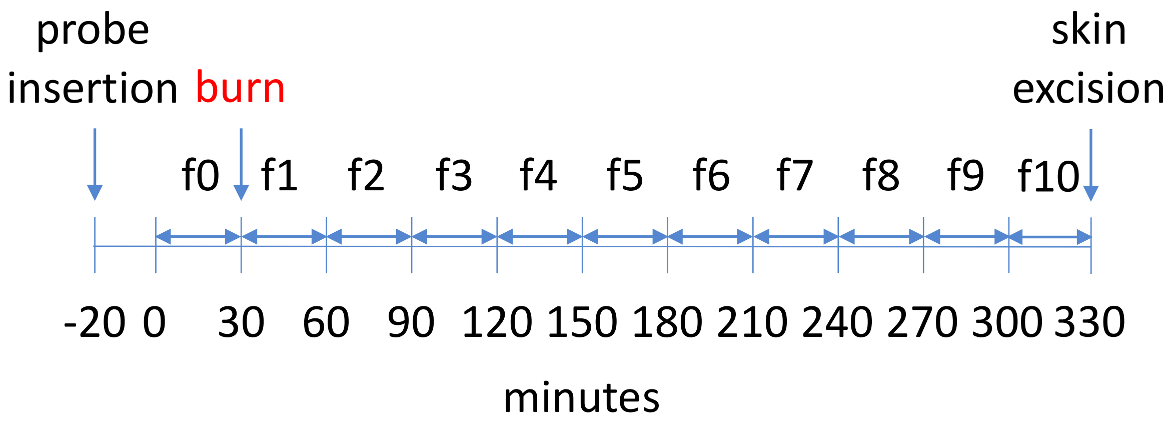

Figure S2. Time-course of the experiment
Sterile microdialysis probes were inserted into subcutaneous tissue of the dorsal aspects of the both hind paws 20 minutes before starting perfusion at 0 minutes. The first microdialysate fractions (f0) were collected between 0 and 30 minutes from both sides. At 30 minutes timepoint, burn injury was induced by immersing one of the paws into 60oC water for 2 minutes. After the injury, microdialysate fractions 1-10 (f1-10) were collected at 30 minutes interval again from both sides. Three hours after the injury the microdialysis was stopped and the skin was excised from both paws.

Disease Models & Mechanisms • Supplementary information

Table S1. Differentially expressed genes in the skin three hours following burn injury.

Click here to Download Table S1

Table S2. GO terms involving or indirectly related to cytokine function in gene sequencing comparison of burn and control tissue.

Click here to Download Table S2
